# Network statistics of genetically-driven gene co-expression modules in mouse crosses

**DOI:** 10.3389/fgene.2013.00291

**Published:** 2013-12-26

**Authors:** Marie-Pier Scott-Boyer, Benjamin Haibe-Kains, Christian F. Deschepper

**Affiliations:** ^1^Cardiovascular Biology Research Unit, Institut de Recherches Cliniques de MontréalMontreál, QC, Canada; ^2^Bioinformatics and Computational Genomics Research Unit, Institut de Recherches Cliniques de MontréalMontreál, QC, Canada

**Keywords:** genetics, network inference, mouse recombinant inbred strains, gene co-expression modules, chromosome domain

## Abstract

In biology, networks are used in different contexts as ways to represent relationships between entities, such as for instance interactions between genes, proteins or metabolites. Despite progress in the analysis of such networks and their potential to better understand the collective impact of genes on complex traits, one remaining challenge is to establish the biologic validity of gene co-expression networks and to determine what governs their organization. We used WGCNA to construct and analyze seven gene expression datasets from several tissues of mouse recombinant inbred strains (RIS). For six out of the 7 networks, we found that linkage to “module QTLs” (mQTLs) could be established for 29.3% of gene co-expression modules detected in the several mouse RIS. For about 74.6% of such genetically-linked modules, the mQTL was on the same chromosome as the one contributing most genes to the module, with genes originating from that chromosome showing higher connectivity than other genes in the modules. Such modules (that we considered as “genetically-driven”) had network statistic properties (density and centralization) that set them apart from other modules in the network. Altogether, a sizeable portion of gene co-expression modules detected in mouse RIS panels had genetic determinants as their main organizing principle. In addition to providing a biologic interpretation validation for these modules, these genetic determinants imparted on them particular properties that set them apart from other modules in the network, to the point that they can be predicted to a large extent on the basis of their network statistics.

## Introduction

In recent years, new technologies such as microarrays have made it possible to generate large numbers of gene expression datasets. To understand how genes interact with one another, methods have been developed to construct gene co-expression networks, and then identify modules of highly connected genes. “Weighted Gene Co-expression Network Analysis” (WGCNA) is the most established and widely used of such methods (Langfelder and Horvath, 2008). Several studies have used these methods to construct (on the basis of gene expression datasets) gene co-expression networks, and then identify modules of highly connected genes (Califano et al., 2012; Cho et al., 2012; Weiss et al., 2012). One common premise of such analyses is that co-expressed genes within modules are more likely to share biological functions. Accordingly, it has been reported several times that some modules detected by gene co-expression analysis show enrichment for genes originating from a particular biologic pathway (Gargalovic et al., 2006; Yang et al., 2009; Rhinn et al., 2013).

The properties of gene co-expression modules can be analyzed in several ways. Eigengenes are values that represent the first principal component of all expression profiles in modules. When networks are constructed using expression data from individuals in a genetic cross, genetic mapping can be performed to test whether the eigengenes of modules show linkage to quantitative trait loci (QTLs), the latter being called “module QTLs” (mQTLs). For instance, mQTLs have been detected in some mouse F2 genetic crosses, with some of them having profiles matching that of phenotypic QTLs (Davis et al., 2012; Leduc et al., 2012). Such findings suggest that the same genetic determinants may link to both a phenotype and the expression levels of genes within the associated module. This suggests that genetic linkage, rather than function, may contribute to coexpression modules detected in genetic crosses However, it is currently not known whether the contributions of genetic determinants to gene co-expression modules represent a common phenomenon, and/or whether corresponding modules have distinctive properties.

Recombinant inbred strain (RIS) are organisms derived from the progenies of crosses of parental inbred strains, and where recombination events between parental chromosomes have been made permanent by long-term inbreeding. When tissue gene expression is measured in RIS by using several animals per strain (to provide both biologic and technical replicates), genetic variations constitute the main cause of variance in gene expression level. Moreover, RIS are homozygous at all loci, which maximizes the potential effect of genetic variation on gene expression. Panels of RIS therefore constitute sensitive backgrounds to study links between genomic variants and gene expression. To test to which extent genomic variants may link to coordinate gene expression within gene co-expression modules, we analyzed publicly available gene expression datasets obtained in several tissues from two kinds of mouse RIS panels. In such panels, we found that a sizeable proportion of gene co-expression modules showed linkage to mQTLs. Moreover, such modules had network statistics that set them apart from other modules in the network. Lastly we observed that these network statistics are sufficiently discriminative to predict, solely on the basis of gene expression, which modules are likely to be genetically-driven.

**Network Inference**

- Type of Biological Networks
  The analyzed networks correspond to gene-co-expression networks constructed from gene expression data obtained in mouse genetic crosses, where genetic variants are the main cause of gene expression variance.
- Utility of the Inferred Networks
  We focused on the detection of gene co-expression network modules showing linkage to quantitative trait loci in multiple independent datasets. We tested the reproducibility of our findings across multiple datasets and across two network inference methods.
- Summary of Results
  In tissues from mouse recombinant inbred strain (RIS) panels, a sizeable portion of gene co-expression modules had genetic determinants as their main organizing principle. These modules had particular properties that set them apart from other modules in the network, to the point that they can be predicted on the sole basis of their gene expression profile characteristics and associated network statistics.

## Materials and methods

### Datasets preprocessing

Discovery datasets were used to test whether gene co-expression modules showing linkage to mQTLs had properties and network statistics that set them apart from other modules. In follow-up experiments, validation sets were used to test whether the properties and network statistics of gene co-expression modules (as determined in the validation sets) could be used to predict accurately whether gene co-expression modules corresponded to a particular type of modules. The discovery sets comprised data obtained in five tissues and one purified cell population from BxD mouse RIS, as well as one tissue from AxB/BxA mouse RIS (Table 1). The validation sets comprised data obtained in one purified cell population from BxD and one tissue from AxB/BxA mouse RIS (Table 1). All data were obtained from the www.genenetwork.org web site, and comprised both gene expression data as well as genomic maps. For gene expression analysis, we used for each gene the one single probe that corresponded to the most variant one. To reduce computation time and facilitate the comparisons between networks, we used the data for the 20,000 most variant genes in each tissue (corresponding to the number of genes that was the smallest common denominator among all datasets used).

**Table 1:** **Gene expression datasets from tissues of mouse RIS used for either discovery or validation analyses in the present study**.

**Discovery datasets**	**Mouse RIS panel**	**Tissue**	**Microarray platform**	**# of WGCNA modules total/gen**	**# of GeneNet modules total/gen**
GN373	24 AXB-BXA	Liver	Affy	95/10	313/31
GN207	68 BXD	Whole eyes	Affy	49/11	42/16
GN160	47 BXD	Lung	Affy	42/12	124/34
GN389	48 BXD	Pituitary	Affy	52/15	65/21
GN122	33 BXD	Regulatory T cells	Affy	77/11	311/34
GN260	38 BXD	Spleen	Illumina	45/13	177/52
*GN323*	*46 BXD*	*Brain amygdala*	*Affy*	*34/0*	*168/32*
**VALIDATION DATASETS**					
GN210	24 AXB-BXA	Whole eyes	Illumina	43/4	74/6
GN319	31 BXD T cell helper	Helper T cells	Affy	68/12	280/39

First column: GeneNetwork ID number of the dataset. Second column: type of mouse RIS and number of strains used in the study. Third column: name of tissue or type of cell used. Fourth column: microarray platform used in the study. Fifth column: number of gene co-expression modules (total and “type 1” genetic) detected in each network using WGCNA for construction of the network. Since no genetic module was detected in dataset GN323 using default parameters, this dataset was not used for analysis of WGCNA modules. Sixth column: number of gene co-expression modules (total and “type 1” genetic) detected in each network using GeneNet for construction of the network.

### Network construction and modules detection

We used the “Weighted Gene Co-expression Network Analysis” (WGCNA) R package (Langfelder and Horvath, 2008) to construct the gene co-expression networks. To avoid computationally intensive tuning of WGCNA parameters, we used all default parameters as proposed previously (Zhang and Horvath, 2005). Within a network, each gene represents a node, and the connections between nodes are defined as edges. To obtain comparable networks between the different datasets, we utilized the top 25% most significant edges in each network. To define modules (i.e., clusters of highly interconnected genes), we used the dynamic tree cut algorithm implemented in the dynamicTreeCut function. “Eigengenes” are summary values representative of the gene expression profiles in corresponding modules. Accordingly, eigengene values can be used to detect “module-QTLs” (mQTLs), i.e., QTLs showing linkage to entire gene co-expression modules(Davis et al., 2012; Leduc et al., 2012). For each module, we used WGCNA to calculate its corresponding eigengene value, and performed QTL mapping with the “R-QTL” tool (Broman et al., 2003), using a detection threshold corresponding to a “logarithm-of-the-odds” (LOD) score of 3.3 (Lander and Kruglyak, 1995). Modules shown for illustration were drawn using the Cytoscape software (Shannon et al., 2003).

In order to test the robustness of our findings with respect to the network inference approach, we also used the GeneNet R package (Schaefer et al., 2006) to construct the gene co-expression networks. This method uses partial correlation to calculate the link between two genes and has the advantage of not requiring any parameter (with the exception of the correlation threshold used to select the most relevant edges). The results derived from GeneNet are reported in Supplementary Information.

### Comparisons between modules

To estimate the contribution of each chromosome to a module, we calculated the percentage of genes that each chromosome contributed to the module. The one chromosome with the highest percentage was considered as the “top contributing” chromosome, and the corresponding percentage value was considered as the “enrichment index for single chromosome contribution.” To calculate a normalized index (and thus allow comparisons across modules), the enrichment index value was divided by the mean of the percentages of genes contributed by all other chromosomes in the module.

Each module was also characterized in terms of its “network statistics” (also known as “fundamental network concepts”) (Dong and Horvath, 2007). We thus calculated the values of heterogeneity, centralization, and density, using the function “fundamentalNetworkConcepts” of WGCNA R package (Langfelder and Horvath, 2008). Comparisons between groups were performed using either the non-parametric Wilcoxon Signed Rank test (for binary comparisons) or the Kruskal Wallis test (for comparisons involving more than 2 classes). Combined *P*-values were calculated using the Z transform approach (Whitlock, 2005), using the survcomp R package (Schröder et al., 2011).

### Validation tests

In the datasets used for validation (Table 1), we first calculated the values of heterogeneity, centralization, density and normalized enrichment index in order to identify which modules could be considered as being “genetically-driven” (according to our own definition: see below). We then ranked all modules according to corresponding values by grouping them in “top percentile” windows ranging from the top 5% to the top 80% (in successive 5% steps). We then: (1) tested whether modules in the top percentile windows corresponded or not to genetically-driven modules, and (2) calculated the accuracy with which each network statistic value categorized corresponding modules. For the latter tests, we calculated the numbers of modules whose characteristics were truly positively predicted (TP), truly negatively predicted (TN), falsely positively predicted (FP) and falsely negatively predicted (FN), and we calculated the receiving operating characteristics (ROC) curves based on sensitivity and specificity, using the ROCR package in R.

All network statistics (heterogeneity, centralization, density and normalized enrichment index) were analyzed independently.

## Results:

### Genetically-linked and genetically-driven modules

**Figure 1:**
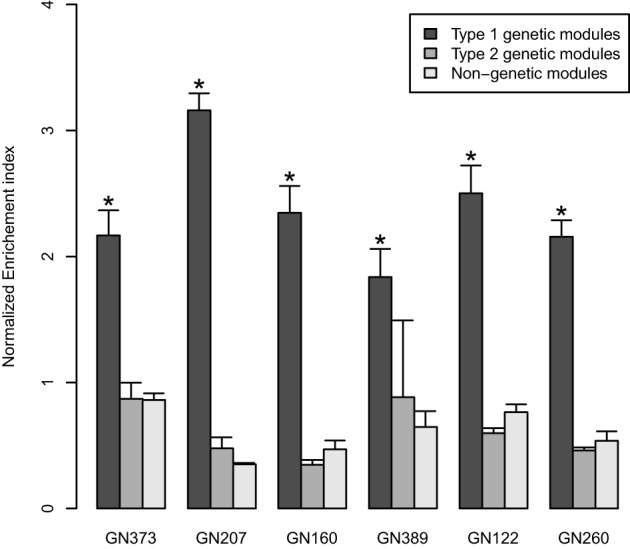
**The bar graphs represent normalized enrichment indices (mean ± SD) in the 6 tested discovery datasets.** The indices quantify to which extent genes in co-expression network originate from a single chromosome. Black bars: values for “genetically-driven” modules (type 1 genetic modules); gray bars: values for the other “genetic” modules (type 2); white bars: values for “non-genetic modules.” ^*^P < 0.05 (Kruskal Wallis tests).

Gene co-expression networks were built using WGCNA for seven RIS mouse expression datasets (Table 1). Since the datasets were obtained using different microarray platforms for different tissues from different animal crosses, we built gene co-expressions network using the same number of genes (the 20,000 most varying genes) and selected the 25% most significant edges in the networks. This approach allowed us to generate networks with comparable characteristics. For each network, we extracted modules containing at least 30 genes, and found that networks contained in average 56 modules (Table 1). Genomic mapping analyses were performed for the eigengenes of all modules to determine whether we could detect linkage of modules to mQTLs. We found that in 6/7 networks, we could detect modules that could be considered as “genetically-linked,” on the basis of showing linkage to a mQTL. In these 6 networks, the proportion of such genetically-linked modules averaged 29.3% (sd 8.4%) (with values ranging from 15.7 to 36.7%)., could be. For 74.6% of these genetically-linked modules, the chromosome harboring the mQTL corresponded to the top-contributing chromosome. Since in such cases the location of the mQTL corresponded to the chromosome that contributed most genes to the modules, we considered these particular modules to be “genetically-driven.” In further comparisons, we called such modules “type 1 genetic modules”; genetically-linked modules where the top-contributing chromosome was not the same as the one harboring the mQTL were called “type 2 genetic modules.” For both types of genetic modules, we calculated the “normalized enrichment index for single chromosome contribution,” and compared it to that of other modules that did not show linkage to any mQTL (“non-genetic modules”) (Figure 1). In all 6 tested WGCNA networks, normalized enrichment index of type 1 genetic modules was significantly higher than that of other types of modules, with type 2 genetic modules showing no difference in comparison to non-genetic modules (Figure 1).

### Network statistics

**Figure 2:**
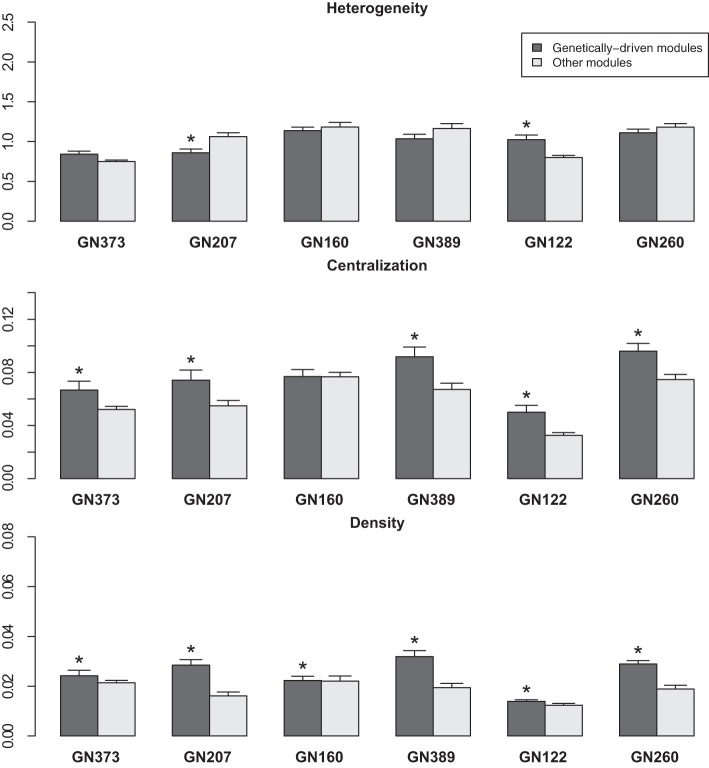
**The bar graphs represent the heterogeneity, centralization and density values (mean ± SD) of modules within networks from the 6 tested discovery datasets.** Black bars: “genetically-driven” modules; gray bars: other modules. ^*^P < 0.05 (Wilcoxon Signed Rank test).

For further analyses, we studied the three following network statistics (Dong and Horvath, 2007): (I) density (which corresponds to the mean connectivity of the network); (II) centralization (which takes the value 0 if the network has a star topology and the value 1 if all nodes have the same connectivity); and (III) heterogeneity (which is the coefficient of variation of the connectivity of the network). Within each studied network, we calculated these three values for genetically-driven (type 1 genetic) modules, and compared them to that obtained other modules in the network (including both the type 2 genetic and the non-genetic modules) (Figure 2). Density was significantly higher (*P* < 0.05) in genetically-driven modules for all six networks, whereas centralization was significantly higher in genetically-driven modules for 5 out of 6 of the studied networks (Figure 2). We did not observe a consistent trend for heterogeneity (Figure 2). When all six modules were combined to calculate overall *P*-values, the differences between type 1 genetic modules vs. all other modules were significant for centralization (p = 9.68 e-06) and density (p = 2.02 e-08), but not for heterogeneity (*p* = 0.457). Differences in network statistics were not due to differences in the sizes of the modules since the latter showed no significant difference in genetically-driven networks compared to the other modules.

**Figure 3:**
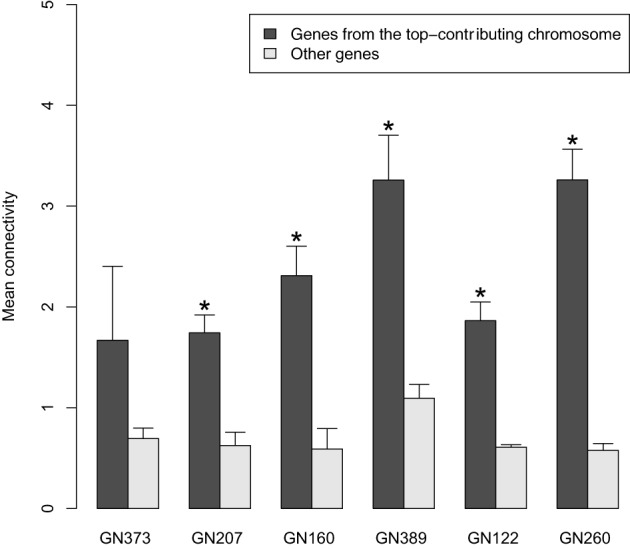
**Comparisons (for the genetically driven modules detected in the 6 tested discovery datasets) of the mean connectivity values of genes originating from the top-contributing chromosome vs. that of other genes in the modules.** The bars represent mean ± SD. ^*^P < 0.05 (Wilcoxon Signed Rank test).

Given that (I) density was higher in genetically-driven modules; and (II) these modules showed enrichment in genes originating from one single chromosome, we tested in these modules whether the connectivity of genes from the top-contributing chromosome was higher than that of other genes in the modules We found that this was indeed the case, with differences being significant for genetically-driven modules in 5 out of the 6 networks tested (Figure 3). When all datasets were combined, the overall *P*-value for connectivity was 5.8e-18.

### Validation tests

**Figure 4:**
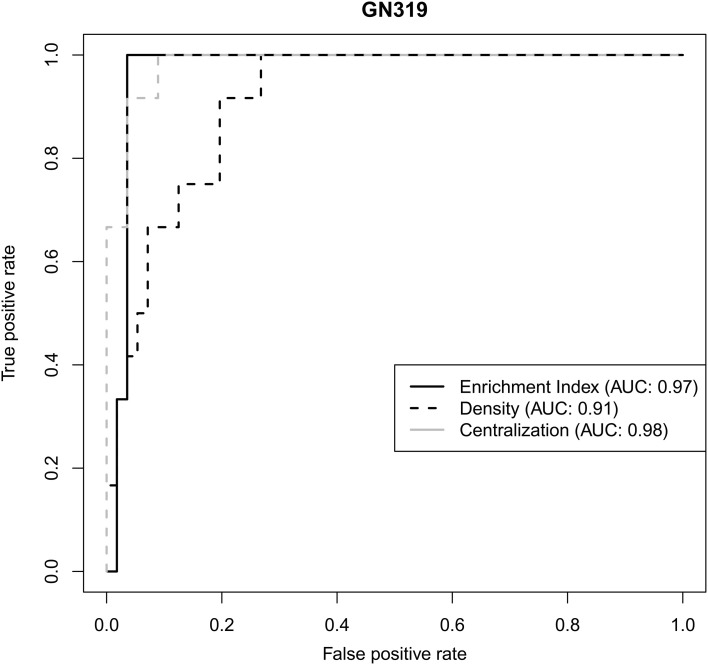
**Receiver operating characteristic (ROC) curves illustrating how 3 different network statistics discriminate genetically-driven modules from other modules in a validation set**.

We used two independent validation datasets to test how robustly network statistics values could discriminate genetically-driven modules from the other ones. In the GN319 dataset, the “area under the curve” (AUC) values for ROC curves were all higher than 0.9, with normalized enrichment index and centralization being most predictive (Figure 4). Even in GN210 (where the proportion of type 1 genetic networks was <10%), network statistics still had good predictive power, since all AUC values were greater than 0.7 (data not shown).

### Alternative network inference method

**Figure 5:**
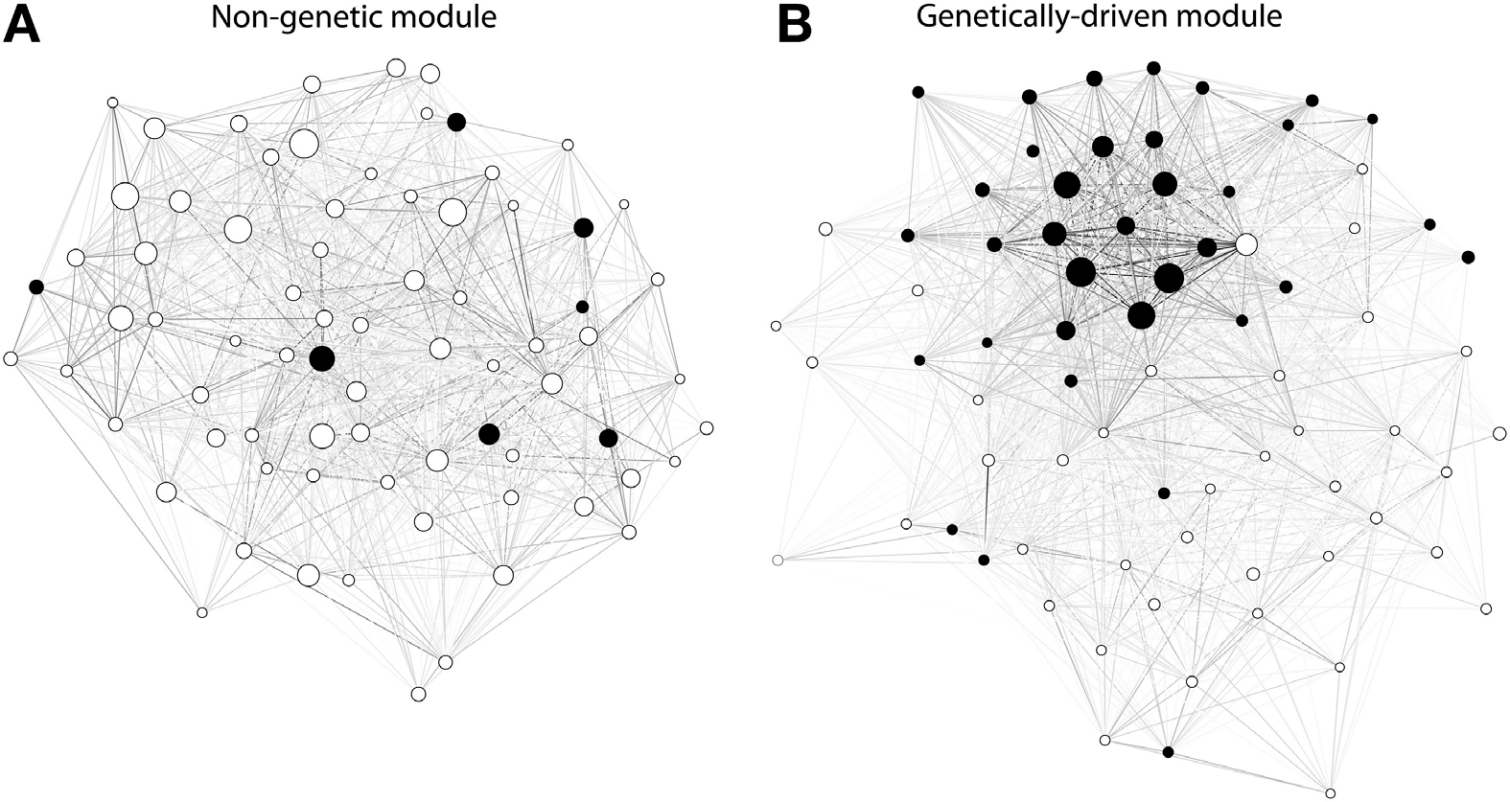
**Ilustrative examples of gene expression modules detected in the GN122 dataset from regulatory T cells (on the basis of the gene co-expression network being built using GeneNet).** Each module was of equal size as they both contained a total of 75 genes; **(A)**: non-genetic module; **(B)**: genetic module. Each node is represented by a circle, either full (when the corresponding gene originates from the top contributing chromosome) or empty (other genes). The edges are colored according to a gray scale, where the darkness of the edge is proportional to the connectivity between 2 nodes. It can be seen that the genetically-driven module contains a higher number of genes from the top-contributing chromosome. Moreover, that module contains a core a several genes displaying connectivity levels that are much higher than other genes in the module, which corresponds to the fact that the values of density and centralization were higher in genetically-driven modules.

To test the robustness of our findings we performed the analyses previously described using GeneNet (Schaefer et al., 2006) as an alternative method to build networks of gene co-expression. Interestingly, whereas the number of modules detected in the WGCNA networks averaged 60 (*sd* = 21), we detected a higher number of modules averaging 172 (*sd* = 118) in the corresponding networks built using GeneNet, although this difference was not significant (*p*-value = 0.06 by two-sided paired Wilcoxon signed rank test). Nonetheless, regardless of the method used for network inference, our observations concerning the differences between genetic and non-genetic modules held true (with in addition heterogeneity also being significantly higher in genetically-driven modules than in non-genetic modules). The various differences in network statistics are further illustrated in two modules of similar sizes detected in the GN122 dataset on the basis of networks constructed with GeneNet (Figure 5)

## Discussion

Complex genetic quantitative traits result from the many interactions of genetic variants with environmental factors, and only a minority of are believed to result from the dysregulation of only one gene (Plomin et al., 2009). Moreover, biological systems are typically organized as modular networks where genes act synergistically rather than representing the sum of their individuals actions (Cho et al., 2012; Weiss et al., 2012). Consequently, gene co-expression network analyses have been proposed as a means to better understand the mechanisms of complex regulatory biologic processes (Califano et al., 2012; Cho et al., 2012; Weiss et al., 2012). Up until now, much of the interpretation of gene co-expression has relied on empirical observations.

For instance, one common strategy has been to rely on annotations (either gene ontology or pathway information) to test whether module show enrichment for genes related to annotated functions. However, the drawbacks are that: (1) “canonical” pathways are often still incomplete, and in fact represent “oversimplifications”; and (2) enrichment analyses are biased toward what we already know (Carro et al., 2010; Farber, 2013).

In some instances, gene co-expression modules have shown linkage to mQTLs in genetic animal crosses, with some of them having profiles matching that of phenotypic QTLs (Davis et al., 2012; Leduc et al., 2012). In such cases, it is likely that a valid biologic process drives gene co-expression in the module. To test to which extent such mechanisms could underlie the organization of gene co-expression modules in genetic crosses, we performed gene co-expression network analyses of datasets originating from eight different tissues and two different panels of mouse RIS. We found (on the basis of detection of mQTLs) evidence of genetic contributions for an average of 29% of the modules. For about 73% of these genetically-linked modules, the influence of the genetic determinants appeared to be even stronger, as the mQTL was located on the same chromosome that was the highest contributor of genes to the module. In such modules, the normalized enrichment index for single chromosome contribution was significantly higher than in other types of modules. Given this clustering of co-expressed genes around mQTLs, we considered such modules as being “genetically-driven.” These modules also appear to have specific features in terms of network statistics: (1) their density was higher, indicating that their mean connectivity was higher than that of other modules; (2) their centralization value was higher, which is compatible with the presence of a core several highly connected genes (in opposition to the presence of one main hub gene regulating all others in the module). Since genetically-driven modules show enrichment for genes originating from one chromosome, these differences in network statistics might be explained if these genes showed higher connectivity than that of other genes in the module. We thus tested this possibility, and found that within genetically-driven modules, connectivity of genes from the top-contributing chromosome was in average 2.25 higher than that of other genes in the module. Our observations did not depend on network inference approaches, as similar conclusions were reached using either WGCNA or GeneNet.

Thus, the gene composition and network statistics of genetically-driven modules indicate that one of their main component is constituted by several highly connected genes originating from one chromosome. In mammals, co-expressed genes have been reported to cluster both at either short-range (1 Mb) or long-range (>10 Mb) levels (Woo et al., 2010). Moreover, we have recently reported in mouse RIS the existence of clusters of co-expressed genes that all show linkage to one common QTL (Scott-Boyer and Deschepper, 2013). Corresponding genomic regions showed a greater abundance of polymorphic SINE retrotransposons, the latter showing enrichment for the motifs of binding sites for various regulators of transcription. We postulate that such mechanisms may account (at least in part) for the presence of several high co-expressed genes within chromosome domains, which constitute the core of gene co-expression modules that have characteristics that set them apart from other kinds of modules.

In mouse RIS, genetically-driven modules are not a rare occurrence, since they constitute in average 21% of all modules. Their network statistics differ substantially from that of other modules, with high AUC values being obtained for the normalized enrichment index as well as the density and centralization valuesThis suggests that genetically-driven modules can, to some extent, be predicted solely on the basis of their gene expression patterns.

In summary, genetic determinants constitute one main organizing principle of a sizeable portion of gene-co-expression modules detected in mouse RIS panels, which provides a biologic validation for corresponding modules. In addition, these modules appear to derive from cores of highly inter-connected genes clustering on one chromosome. This may constitute one particular mechanism driving gene co-expression, which imparts on genetically-driven modules particular properties. These properties set them apart from other modules in their network, to the point that they can be predicted to a large extent on the basis of their network statistics. Of note, it is possible that RIS panels provide a background that is particularly appropriate for the detection of genetically-driven modules. It remains to be seen to which extent they will be detectable in other types of genetic crosses.

## Author contributions

All authors participated to the writing of the manuscript and design of the experiments, with most analyses having been performed by Marie-Pier Scott-Boyer.

### Conflict of interest statement

The authors declare that the research was conducted in the absence of any commercial or financial relationships that could be construed as a potential conflict of interest.
