# Supplementary material for: Network statistics of genetically-driven gene co-expression modules in mouse crosses
Source: Front Genet. 2013 Dec 26;4:291. doi: 10.3389/fgene.2013.00291 (PMC3872724; doi:10.3389/fgene.2013.00291)
Supplement: Supplementary file 1 [file DataSheet1.PDF]

SUPPLEMENTARY INFORMATION

Network statistics of genetically-driven gene co-expression modules in mouse crosses

Marie-Pier Scott-Boyer, Benjamin Haibe-Kains and Christian F. Deschepper

This Supplementary Information contains the results based on the modules identified by GeneNet as opposed to the results of WGCNA presented in the main text.

Fig S1

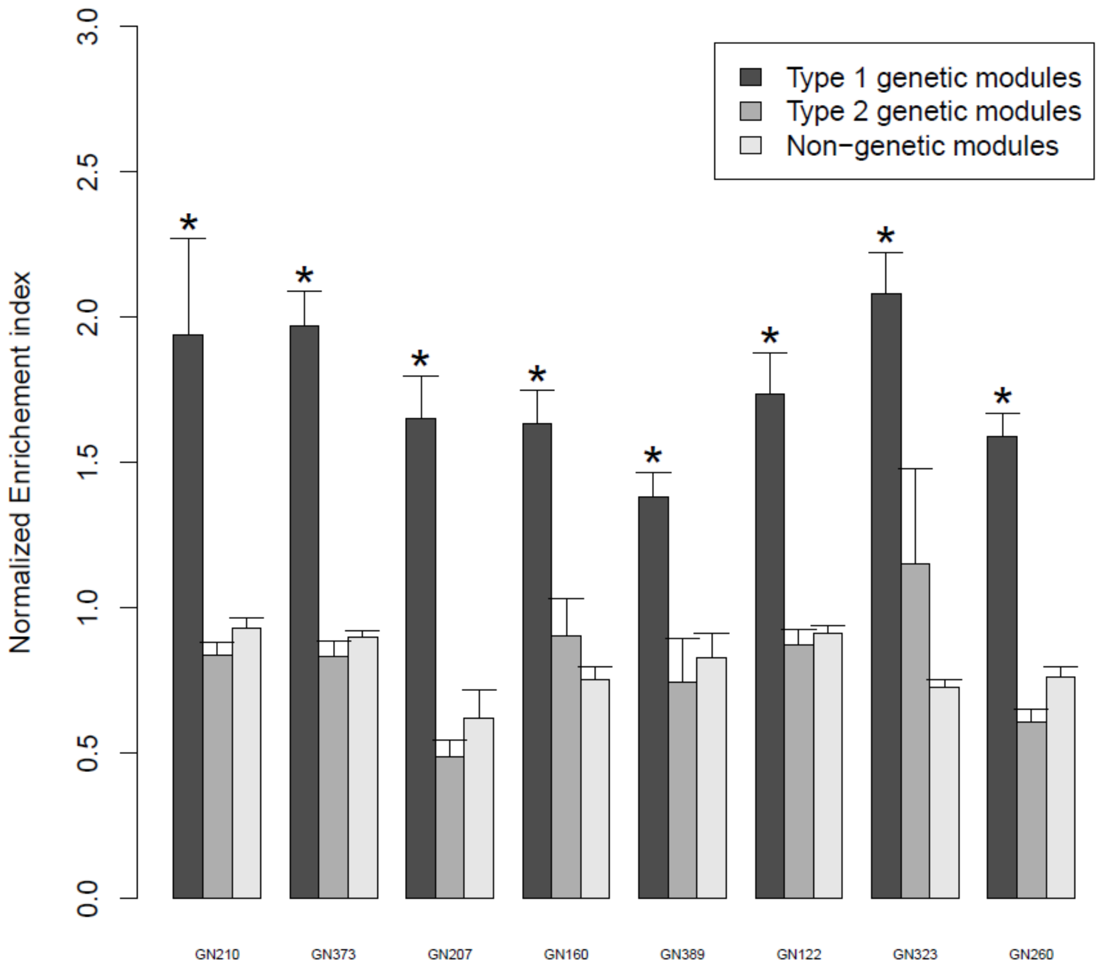

The bar graphs represent normalized enrichment indices (mean ± SD) in the 8 discovery datasets. The indices quantify to which extent genes in co-expression network originate from a single chromosome. <u>Black bars</u>: values for "genetically-driven" modules (type 1 genetic modules); <u>grey bars</u>: values for the other "genetic" modules (type 2); <u>white bars</u>: values for "non-genetic modules". **P* < 0.05 (Kruskal Wallis tests).

Fig S2

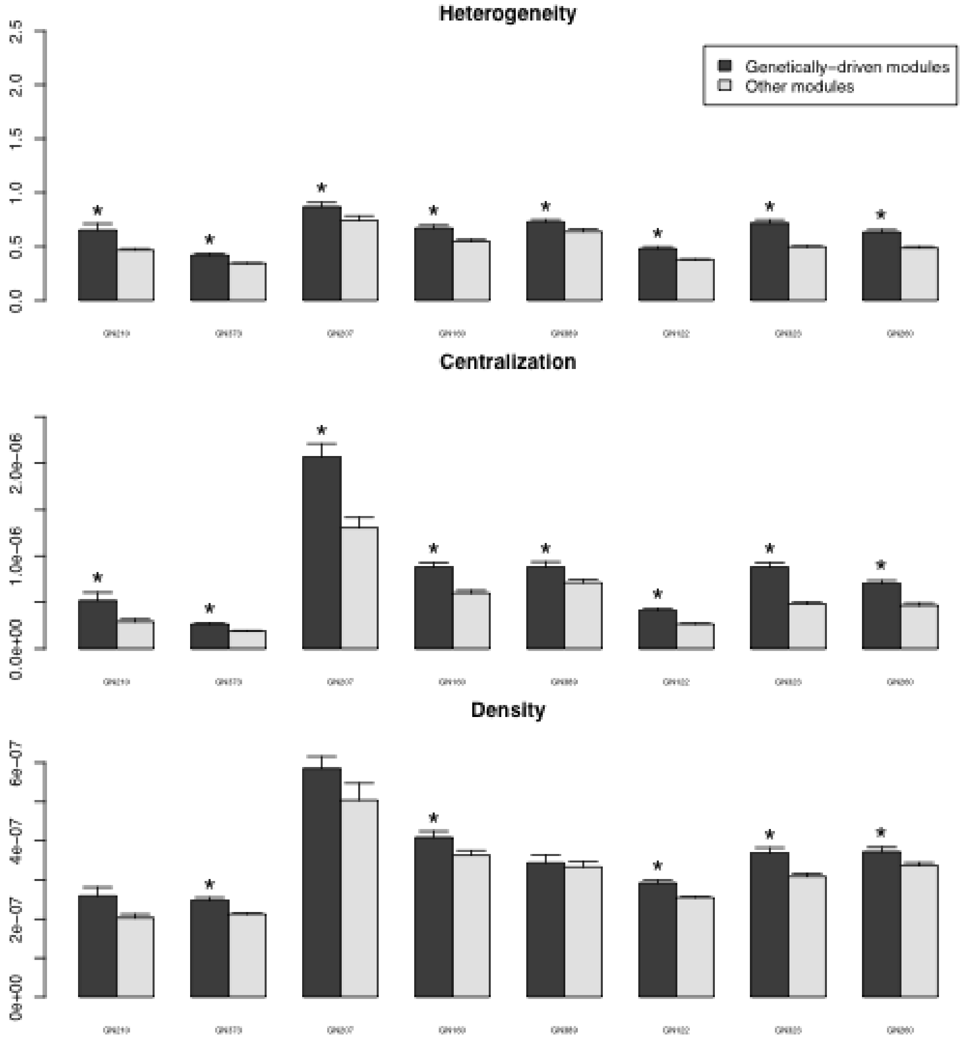

The bar graphs represent the heterogeneity, centralization and density values (mean ± SD) of modules within networks from 8 different dataset. Black bars: "genetically-driven" modules; grey bars: other modules. **P* < 0.05 (Wilcoxon Signed Rank test).

Fig S3

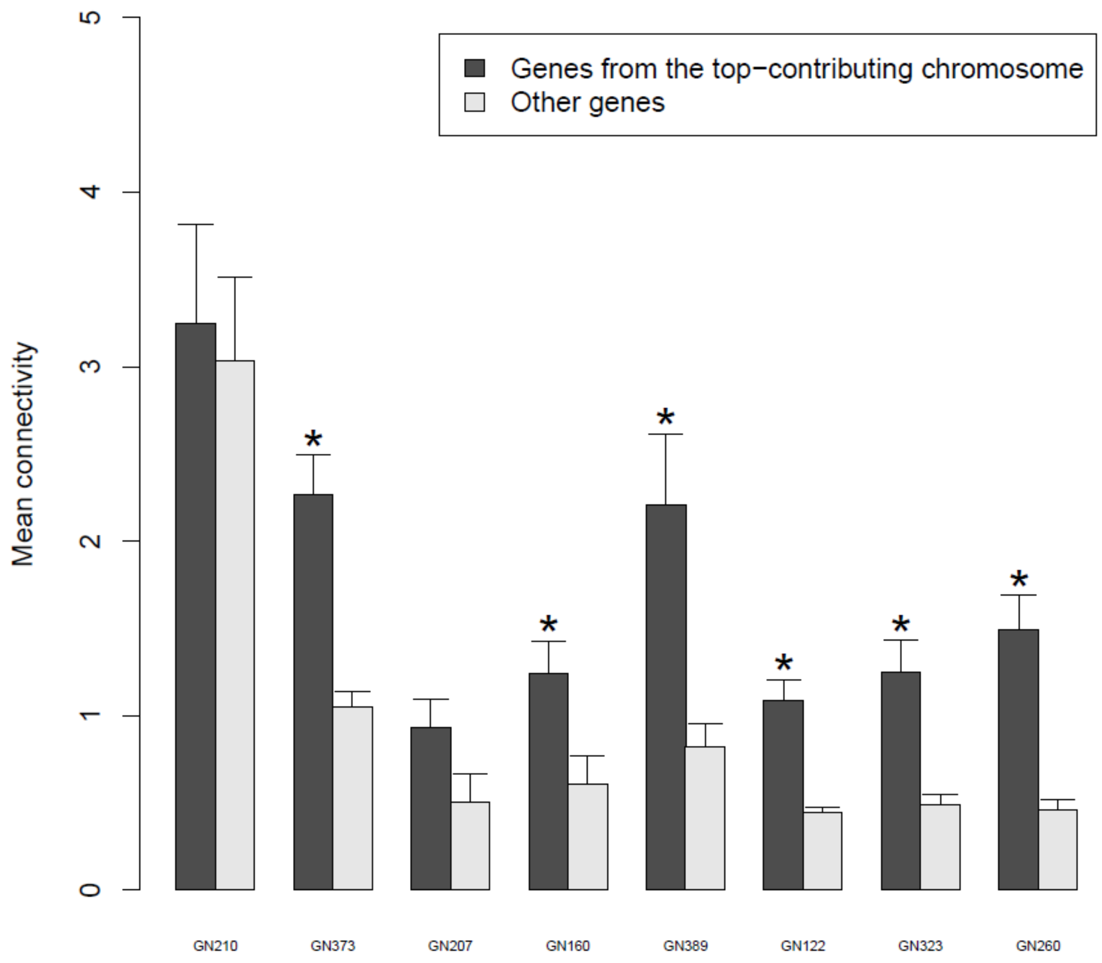

The bar graphs represent (for genetically driven module from 8 different datasets) the mean connectivity values (mean ± SD) of genes originating from the top-contributing chromosome compared with other genes from the network. *$P < 0.05$ (Wilcoxon Signed Rank test).
